# Clinical Features of Patients With Progressive Supranuclear Palsy in an US Insurance Claims Database

**DOI:** 10.3389/fneur.2021.571800

**Published:** 2021-06-17

**Authors:** Emma Viscidi, Irene Litvan, Tien Dam, Maneesh Juneja, Li Li, Henry Krzywy, Susan Eaton, Susan Hall, Joseph Kupferman, Günter U. Höglinger

**Affiliations:** ^1^Biogen, Cambridge, MA, United States; ^2^Parkinson and Other Movement Disorders Center, University of California, San Diego, San Diego, CA, United States; ^3^MJ Analytics Ltd., Hemel Hempstead, Hertfordshire, United Kingdom; ^4^Department of Neurology, Technische Universität München, Munich, Germany; ^5^German Center for Neurodegenerative Diseases (DZNE), Munich, Germany; ^6^Department of Neurology, Hanover Medical School, Hanover, Germany

**Keywords:** movement disorder, natural history, neurodegenerative disease, progressive supranuclear palsy, rare disease

## Abstract

**Background:** Progressive supranuclear palsy is a rare neurodegenerative movement disorder and little is known about its epidemiology.

**Objective:** Estimate age-adjusted prevalence of progressive supranuclear palsy and describe antecedent diagnoses and progressive supranuclear palsy patient features in the 5 years before first diagnostic code.

**Methods:** In a nested case-control study in the IBM MarketScan Commercial and Medicare Supplemental Databases, a large set of US insurance databases containing medical service and prescription drug claims from employer-based commercial and Medicare supplemental health insurance plans, progressive supranuclear palsy cases (identified via International Statistical Classification of Diseases 9th/10th revision codes) and controls were included if enrollment was ≥1 month in the study period (October 1, 2015–October 31, 2017). Two controls with no diagnosis codes for PSP were matched to cases on birth year, sex, enrollment time in the database, and pharmacy benefit eligibility. Controls were assigned a randomly selected index date from their eligibility period. Prevalence of progressive supranuclear palsy was estimated in 2016 among patients with ≥1 month of continuous enrollment in that year. Prevalence ratios for comorbidities (claim/diagnosis codes) were examined in the ≤ 5 years before index date (first progressive supranuclear palsy claim date).

**Results:** Age-adjusted progressive supranuclear palsy prevalence was 2.95/100,000 in 2016. The most common diagnosis codes in cases vs. controls in the 5 years pre-index were gait abnormalities (79.3 vs. 21.8%), pain in joint (54.9 vs. 36.0%), Parkinson's disease (54.6 vs. 1.0%), fatigue (49.8 vs. 21.6%), and cerebrovascular disease (45.6 vs. 16.4%).

**Conclusions:** In this large database analysis, based on preliminary analyses, the prevalence of diagnosed progressive supranuclear palsy was 2.95/100,000, which is lower than many prior studies. Typical symptoms suggestive of progressive supranuclear palsy were present before index date, indicating a potential delay in time to diagnosis. The identification of diagnostic codes for clinical features of progressive supranuclear palsy that occurred before index date may be used to develop predictive models to identify potential progressive supranuclear palsy patients earlier in their disease course.

## Introduction

Progressive supranuclear palsy (PSP) is a rare neurodegenerative movement disorder characterized by motor, behavioral, and cognitive dysfunction (1, 2). The classic clinical features of PSP include gait and balance impairment, axial parkinsonism, frontal dementia, supranuclear vertical gaze palsy leading to impaired vision, hypophonic/spastic speech patterns, and at later stages dysphagia leading to aspiration (2–4). Postural instability is the initial symptom in approximately two thirds of patients with the Richardson syndrome (PSP-RS), the classical PSP phenotype (2); other early presenting features of this phenotype may include slowing of vertical saccades, axial rigidity, or apathy (5, 6). Neuropathological examination of PSP patients reveals aggregates of the protein tau in 4-repeat isoforms in specific brain areas, notably in the basal ganglia, brainstem, deep cerebellar nuclei, and usually frontal neocortex (1, 7–10).

The average age of onset is ~65 years, (11, 12) and death occurs a median of 7.3 years after symptom onset (13). PSP-RS is the most recognized phenotype (1, 5); however, up to half of PSP patients may initially display less specific or well-recognized features of the disease, such as PSP-parkinsonism predominant (PSP-P) (2, 14), and the most common diagnosis at initial assessment is Parkinson's disease (12). Although no disease-modifying treatments are currently available (9, 15–17), a better understanding of the natural history, including comorbidities and early features of PSP is key to patient management.

The prevalence of PSP reported in prior studies ranges from 1.39 to 17.3 per 100,000 persons, with a prevalence of ~6 per 100,000 in Europe (18–22). However, most reports of incidence and prevalence have been from clinical records or regional studies in Europe and Japan (19, 20, 22, 23). Only one study, published in 1988, estimated the prevalence in the United States at 1.39 per 100,000; 50 PSP patients were identified based on a survey of neurologists and chronic care facilities in two New Jersey counties (18). Three other studies examined the incidence of PSP in the United States using data from the Rochester Epidemiology Project, and reported incidence rates ranging from 0.9 to 2.6 per 100,000 (24–26). The present study is the first epidemiologic analysis of PSP using real-world data derived from a large US health insurance claims database. The objectives were to estimate the prevalence of diagnosed PSP and describe the medical conditions and preceding diagnoses of a large sample of PSP patients.

## Materials and Methods

### Data Source

This nested case-control study used data from the IBM MarketScan Commercial and Medicare Supplemental Databases, a large set of US insurance databases containing medical service and prescription drug claims prospectively collected from employer-based commercial and Medicare supplemental health insurance plans. The Commercial database consists of medical and drug data from employers and health plans, encompassing employees, their spouses and dependents who are covered by employer-sponsored private health insurance in the US. The Medicare Supplemental Database includes the Medicare-covered portion of payment (represented as Coordination of Benefits Amount or COB), the employer-paid portion and out-of-pocket patient expenses. The databases protect the privacy of individual patients through synthetic identifiers; all data were fully compliant with the Health Insurance Portability and Accountability Act of 1996. Additional information on the databases can be found at https://www.ibm.com/products/marketscan-research-databases/databases. Institutional review board approval for this study was not required.

### Study Population

PSP cases and controls (patients without PSP) were selected from all patients in the MarketScan databases who had ≥1 months of enrollment from October 1, 2015 through October 31, 2017. This inclusion period was used because a specific diagnostic code for PSP was introduced in the International Statistical Classification of Diseases, 10th (ICD-10) in October 2015. The source population included 38,053,188 total patients in the MarketScan databases, of whom 51% were female, 53% were age <40 years, and 16% were aged 60 years or older. In the source population, 19% of patients were from the Northeast, 21% North Central, 38% South and 22% West of the US.

Cases were identified using a combination of ICD-10 and ICD-9 revision codes. As described above, the ICD-10, which was implemented (i.e., used to code all health claims) in October 2015 in the United States, includes a specific code for PSP [G23.1, progressive supranuclear ophthalmoplegia (1)]. The ICD-9 (used previously in the United States) did not have a specific code for PSP; instead, the code 333.0 (other degenerative diseases of the basal ganglia) (27) was primarily used to document PSP. ICD-9 code 356.8 (other specified idiopathic peripheral neuropathy) may also have been used by clinicians as it includes reference to supranuclear paralysis, but it lacks the specificity to be used for identification of PSP patients and so was not used in this analysis. Cases were required to have at least one ICD-10 diagnostic code for PSP.

PSP cases were defined as those patients enrolled ≥1 months of enrollment from October 1, 2015 through October 31, 2017 aged ≥40 years at index date (defined as the date of the first diagnosis code for PSP), who had either two or more diagnosis codes of ICD-10 code G23.1, or one or more ICD-9 code 333.0 and one or more ICD-10 code G23.1 (inpatient or outpatient), occurring ≥1 month apart. Two diagnostic codes were required to reduce the risk of erroneously including patients with a ‘rule-out' code, that is, patients with one diagnostic code only who were examined for possible PSP but were unlikely to be diagnosed. There were no further exclusion criteria.

Two controls with no diagnosis codes for PSP were randomly selected for each case to draw a sample representative for the reference population; the two groups were matched on birth year, sex, enrollment time in the database, and pharmacy benefit eligibility. Matching by race/ethnicity was not conducted due to considerable missing data on these variables. Controls were assigned a randomly selected index date from their eligibility period. Enrollment in the database refers to the period when a patient was enrolled in the claims database and had health insurance coverage.

Comorbidities were examined in the time period prior to index date from January 1, 2011–October 31, 2017). The comorbidities were classified with the Clinical Classification Software, which was developed for the Agency for Health Research Quality and which groups ICD-9/10 codes into clinically meaningful categories. The frequency of individual ICD-9 and 10 codes within comorbidity categories was summarized (28).

### Statistical Analysis

Prevalence of PSP was estimated for 2016 among patients with ≥1 month of continuous enrollment in the database in that year. This prevalence estimate was directly standardized to the age of the US population, based on the 2010 census. Age-specific prevalence was calculated based on the number of patients in each age group. To examine the occurrence of comorbidities and diagnoses in the time period before index date, claims data predating index date by ≤ 5 years were included in the analysis. Prevalence ratios (PRs) for comorbidities were examined for ≤ 5 years before index date (by year). PRs were calculated by dividing the percentage of cases by the percentage of controls for any given comorbidity category, with a greater PR indicative of a large difference in the frequency between cases and controls. Statistical significance was calculated by 95% confidence intervals (CIs; two-sided *P*-value significance level of 0.05), comparing prevalence in cases and controls. SAS software package was used for all analyses.

## Results

Of the ~38 million patients in the MarketScan databases with ≥1 month of enrollment time in the database from October 1, 2015 through October 31, 2017, 630 patients met the criteria for PSP. The first ICD code of PSP was recorded most frequently by neurologists (44.3%), acute care hospitals (18.8%), and family practice physicians (11.1%; Supplementary Table 1). Men comprised 53.5% of cases (Table 1) and the age distribution by sex was similar among patients with PSP. The mean age at index date was 71.9 (range, 42–96) years and was similar in men and women; most (68.7%) patients were aged 60–79 years.

**Table 1:** Demographics of PSP cases and non-PSP controls.

	**PSP Cases (*N* = 630)**	**Non-PSP Controls (*n* = 1246)**
Sex	*N* (%)	*N* (%)
Male	337 (53.5)	674 (54.1)
Female	293 (46.5)	586 (47.0)
Age at index date, years^a^		
Mean	71.9	71.9
Median (range)	73 (42–96)	73 (42–96)
Age category at index date, years, *n* (%)		
40–49	5 (0.8)	10 (0.8)
50–59	51 (8.1)	102 (8.2)
60–69	197 (31.3)	394 (31.6)
70–79	235 (37.3)	470 (37.7)
80–84	89 (14.1)	178 (14.3)
≥85	53 (8.4)	106 (8.5)
Enrollment time before index date, *n* (%)^b^		
Any time window, ≥= 30 days	590 (94.7)	1,220 (97.9)
1 day to 12 months	477 (76.6)	969 (78.0)
13 to 24 months	394 (63.2)	780 (62.6)
25 to 36 months	303 (48.6)	583 (46.8)
37 to 48 months	188 (30.2)	358 (28.7)
49 to 60 months	60 (9.6)	121 (9.7)

a *The index date is the date of the first claim for PSP for PSP cases and a randomly selected index date from their eligibility period for controls.*

b *Number of patients enrolled during the specified time period prior to index date. Includes 623 PSP cases for whom a matched control was identified. PSP, progressive supranuclear palsy*.

### Prevalence

**Figure 1:**
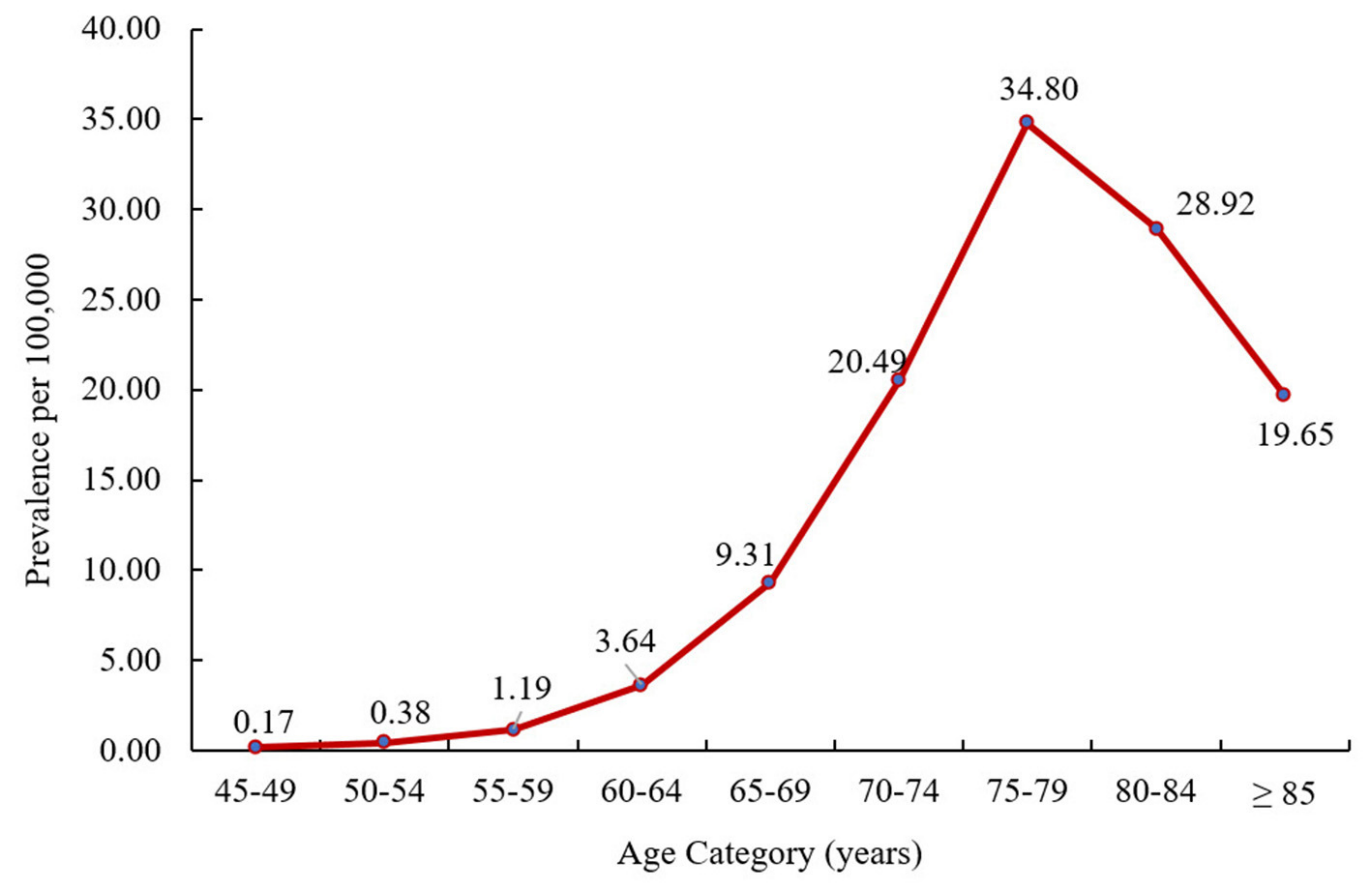
Crude prevalence of PSP by age at PSP index date in 2016. PSP, progressive supranuclear palsy.

PSP patients who had ≥1 month of continuous enrollment in 2016 (*n* = 563) were included in the prevalence analysis (Supplementary Figure 1). The crude all-age prevalence of PSP was 1.89 per 100,000 persons in 2016. The age-adjusted prevalence (standardized to the 2010 US population) was 2.95 per 100,000 persons. The crude prevalence of PSP increased with age, with a peak prevalence of 34.80 per 100,000 in persons aged 75–79 years (Figure 1).

### Diagnoses Preceding the Index Period

A total of 623 of 630 PSP cases were 1:2 matched to 1,246 controls. For seven PSP cases a matched control was not able to be identified using the matching criteria. Of the remaining seven, one was excluded owing to having <1 month of enrollment time and six were not able to be matched to a control because of the matching criteria. Almost 80% of PSP cases were enrolled in the 1-year period before index date and ~30 and ~10% were enrolled in the 4 and 5 years before index date, respectively (Table 1).

**Figure 2:**
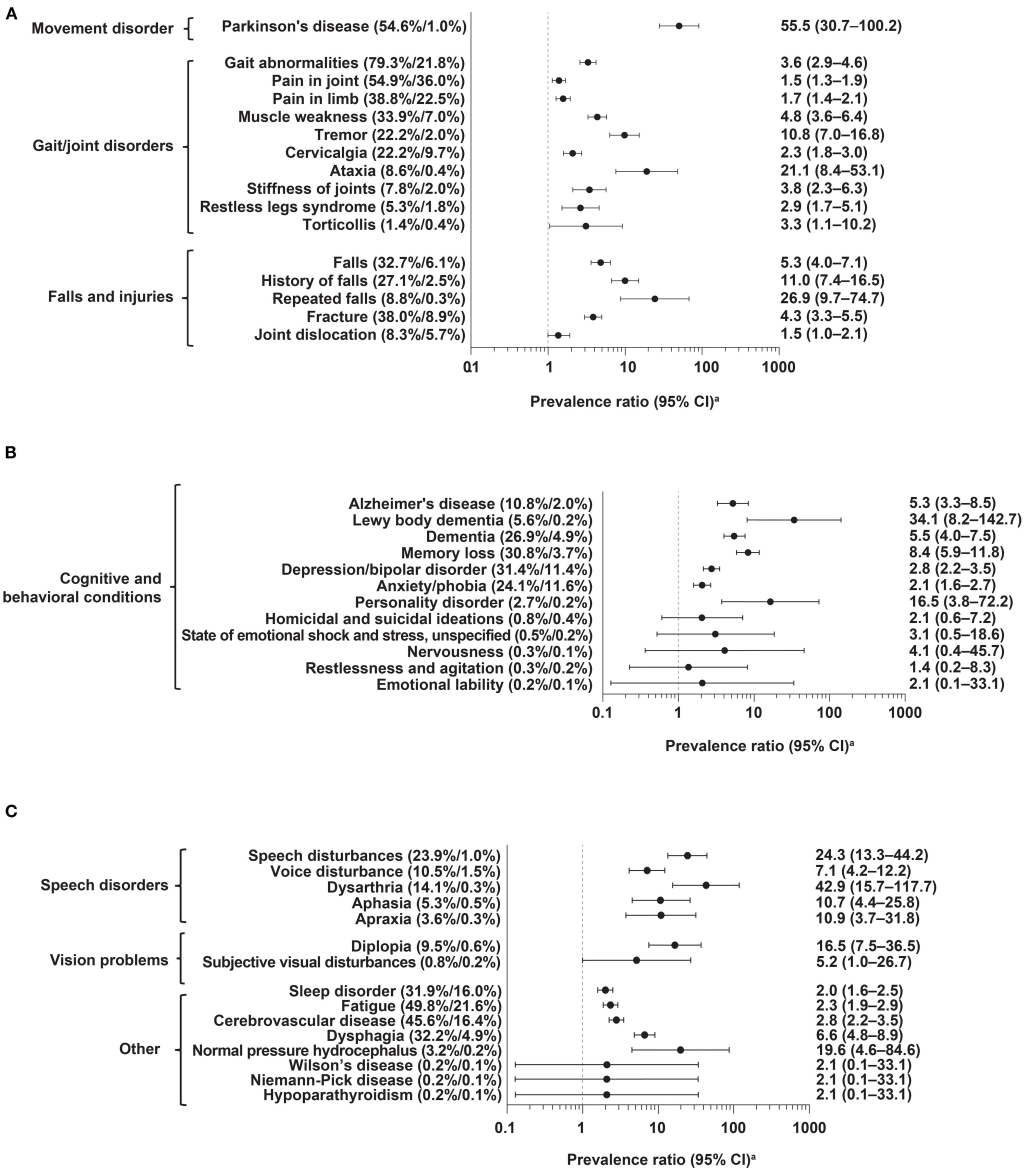
Frequency of diagnoses anytime in the 5 years before index date in PSP cases (*n* = 590) vs. controls (*n* = 1,220). **(A)** Movement disorders, gait/joint disorders, falls and injuries; **(B)** cognitive and behavioral conditions; **(C)** speech disorders, vision problems, other. CI, confidence interval; PSP, progressive supranuclear palsy. ^a^Logarithmic scale.

**Figure 3:**
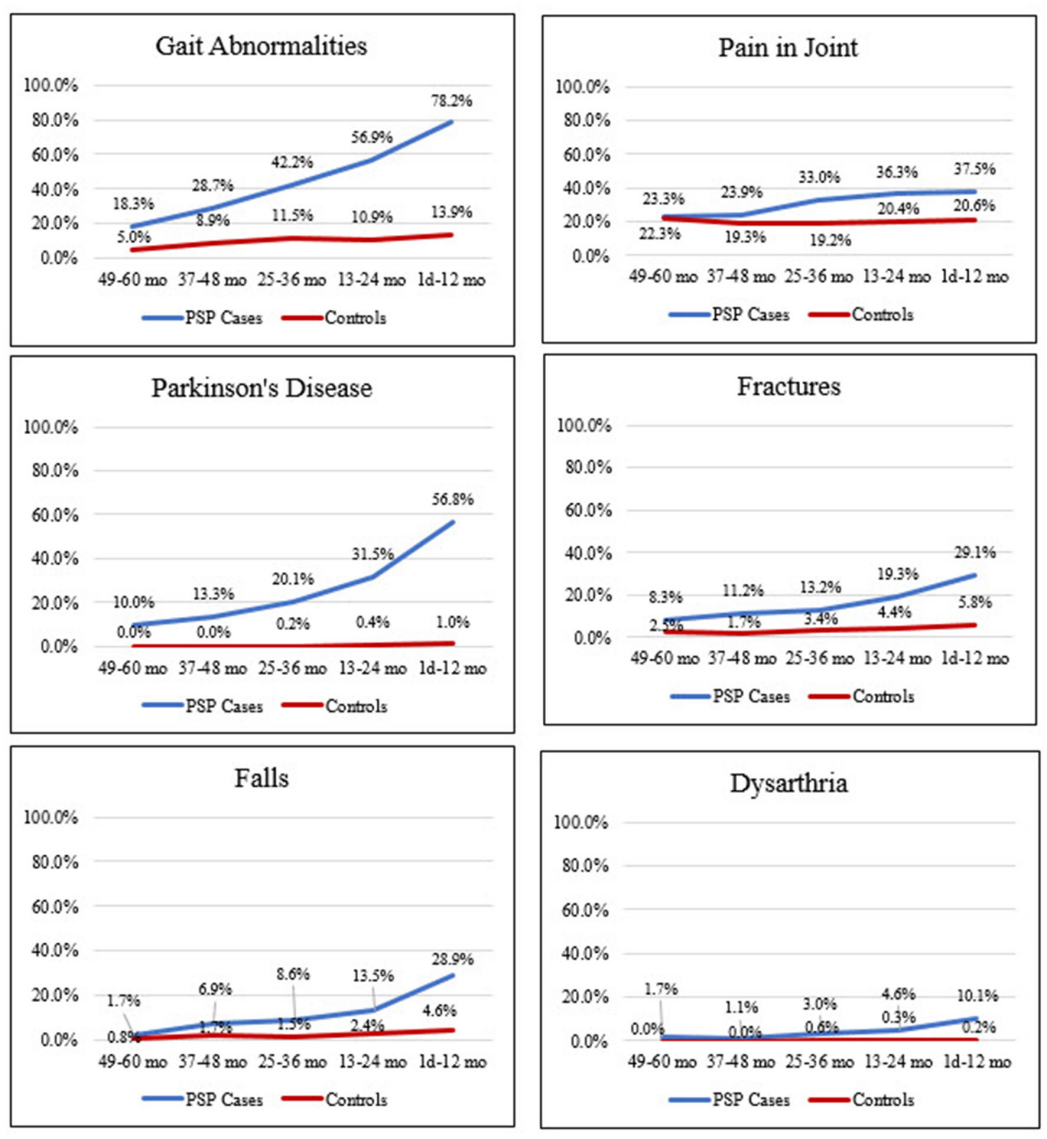
Frequency of clinical features suggestive of PSP in cases and controls in the 5 years before index date. 49–60 months: PSP, *n* = 60; control, *n* = 121. 37–48 months: PSP, *n* = 188; control, *n* = 358. 25–36 months: PSP, *n* = 303; control, *n* = 583. 13–24 months: PSP, *n* = 394; control, *n* = 780. 1 day−12 months: PSP, *n* = 477; control, *n* = 969. Mo, month. PSP, progressive supranuclear palsy.

The most common diagnosis codes in cases (vs. controls) occurring anytime in the 5 years preceding index date were gait abnormalities (79.3 vs. 21.8%), pain in joint (54.9 vs. 36.0%), Parkinson's disease (54.6 vs. 1.0%), fatigue (49.8 vs. 21.6%), and cerebrovascular disease (45.6 vs. 16.4%); all were significantly more common in cases than controls (i.e., lower 95% CI for PR did not cross 1; Figure 2 and Supplementary Table 2). Prevalence of these diagnoses increased in cases during the 5 years before index date but remained relatively stable and generally lower in matched controls (Figure 3 and Supplementary Tables 2, 3).

Potential PSP symptoms, such as fracture, falls, and dysarthria, were significantly more prevalent in cases than controls, occurring at overall frequencies of 38.0 vs. 8.9%, 32.7 vs. 6.1%, and 14.1 vs. 0.3%, respectively (Figure 2 and Supplementary Table 2). These symptoms, as well as other diagnoses examined, also increased in cases during the 5 years before index date, while remaining at relatively stable, lower rates in controls (Figure 3; Supplementary Tables 2, 3 and Figure 2).

Other common diagnoses (≥20% in overall prevalence) in cases with significantly large relative differences in overall frequency vs. controls (as represented by PRs), included speech disturbances (PR: 24.3); history of falls (PR: 11.0); tremor (PR: 10.8); memory loss (PR: 8.4); dysphagia (PR: 6.6); dementia (PR: 5.5); muscle weakness (PR: 4.8); depression and bipolar disorder (PR: 2.8); cervicalgia (PR: 2.3); and anxiety and phobia (PR: 2.1) (Supplementary Table 2).

## Discussion

This nested case-control study, in which PSP cases were compared to controls, provides real-world data from the largest cohort of PSP patients in the world reported to date. The age-adjusted prevalence of PSP was 2.95 per 100,000, which is consistent with, but on the lower end of previously reported estimates of 1.39–6.4 per 100,000 from the US and Europe (18–20, 22, 29). The lower prevalence of PSP observed in this study may be due to the overrepresentation of patients with employer-based health insurance in the database and the underrepresentation of older patients with Medicare insurance. The underrepresentation of older patients would be expected to lower the prevalence estimate, as PSP is known to increase with age (29).

The number of males and females with PSP in our study was similar, and the distribution of PSP by age was similar for men and women. This is in line with other population-based studies that have found no consistent difference in the occurrence of PSP in men and women (19, 20, 24).

The Movement Disorder Society PSP criteria (6) had not yet been published during the study period; therefore, PSP diagnosis in the database most likely reflects the PSP-RS phenotype, which was previously diagnosed based on the National Institute of Neurological Disorders and Stroke diagnostic (NINDS) criteria (5). This study, therefore, reports primarily on PSP-RS, not other subtypes of PSP, and is likely an underestimation of the prevalence of all types of PSP. However, some of the patients in this study may also have PSP-P, which is the second most common variant of PSP and observed in up to 35% of cases (30). In the only epidemiologic study including PSP subtypes other than PSP-RS, conducted in Japan, the prevalence of PSP-RS only was 13.8 per 100,000, while the prevalence of all PSP was higher at 17.3 per 100,000 (21). Potential misdiagnosis may also contribute to the underdiagnoses of PSP in the database: approximately half of PSP patients had a prior PD diagnosis. Furthermore, most clinicians are unaware of PSP and the clinical criteria for diagnosis, resulting in under-diagnosis of the disease. Further studies of PSP in electronic-health record databases, as opposed to claims databases, may allow for the determination of PSP subtype based on provider notes, which could provide better estimates of the true prevalence of PSP in the US general population.

Prevalence of PSP increased with age with a peak at age 75–79 years. Other studies have similarly reported increasing prevalence with greater age with peak rates at ages 60–69 (18), 80–89 (22), and 70–74 years (29). These findings confirm that PSP is a rare, and likely under-diagnosed, condition in the United States, predominantly affecting persons of older age. Countries with aging populations, including the United States, will likely experience an increase in PSP rates over time.

Prior epidemiological studies of PSP in the United States have had mostly sample sizes of ≤ 50 cases from medical institutions in circumscribed regions of the country (18, 24–26). These studies included patients from a very restricted one- or two-county area that often include a regional referral center, thus introducing possible bias toward patients with an advanced or complex condition. Our study draws on national health insurance data, enabling a larger, more geographically diverse population receiving care in a wider range of settings. Additionally, use of a database such as the one in our study, permits longitudinal tracking of patients and facilitates linking of medical and pharmacy claims data to provide a clearer picture of a patient's journey. Although this study of 630 PSP patients is not population based, it provides information on a large sample of PSP patients across the United States. Of the total number of PSP cases identified from commercial and Medicare health insurance plans, 69% had Medicare supplemental plans. Population-based registries are highly valuable in the context of rare neurodegenerative disorders (31), however, there are no such existing registries of patients with PSP in the United States. Therefore, claims databases provided an alternative source for epidemiologic studies of PSP.

This study identified several diagnoses occurring more frequently in PSP cases than controls. Almost half (45.6%) of cases (vs. 16.4% of controls) had a diagnosis code for cerebrovascular disease at some point in the 5 years before diagnosis (PR: 2.8). Although such claims were not based on a validated case definition for cerebrovascular disease, and could also represent a workup for stroke based on presentation of PSP and/or falls, the association of PSP with potential stroke before diagnosis is a relatively new finding that may warrant further research. It is unknown if the identification of diagnostic codes for cerebrovascular disease in this study is indicative of actual diagnoses for cerebrovascular disease or, given that cerebrovascular disease is common and often used as a generic term in patients with vascular spots observed by magnetic resonance imaging (MRI), it may not indicate an actual cerebrovascular disease diagnosis. Vascular PSP is an uncommon variant of PSP characterized by a higher degree of asymmetry, lower body involvement, predominant corticospinal and pseudobulbar signs, urinary incontinence, cognitive impairment, and increased rate of risk factors for stroke compared with other forms of PSP, together with evidence of vascular disease on neuroimaging studies (32). In a study of 128 patients diagnosed with PSP whose records were reviewed for evidence of vascular disease, 30 (23%) had signs consistent with vascular PSP (33). Stroke may also be made as an initial diagnosis in PSP patients. In an epidemiologic study in England, 15/144 (10%) PSP cases were initially diagnosed with stroke by a general practitioner (34). However, a case-control study of 50 PSP patients in the United States did not find that stroke was a risk factor for later-developing PSP (35).

The findings in this study demonstrate that the clinical features of PSP are apparent in patients' medical records several years before first diagnostic code (index date). The most common diagnoses in PSP patients occurring anytime in the 5 years preceding first diagnostic code included diagnostic codes for gait abnormalities, pain in joint, Parkinson's disease, and cerebrovascular disease. Gait abnormalities, cerebrovascular disease, and memory loss were significantly more common in cases than controls in the 4–5 years before index date. At 3–4 years before index date, numerous diagnoses were significantly more common in cases than controls, including dysphagia, history of falls, fractures, Alzheimer's disease, and memory loss. Diagnostic codes for these comorbidities may be useful in the development of predictive models to help identify PSP patients earlier in the disease course.

The findings in this real-world study add to current knowledge on the natural history of PSP, as described in earlier retrospective autopsy and clinic-based studies (3, 34, 36, 37). The diagnostic claims occurring ≤ 5 years before index date in the present study are suggestive of the occurrence and documentation of three of the four functional domains representing the core clinical features of PSP according to clinical criteria and guidelines (5, 6), including postural instability, akinesia, and cognitive dysfunction. The specific vision problems examined in the 5 years before diagnosis showed a low frequency in cases; however, overall eye disorders were frequent in PSP patients, occurring in 85.2% of cases vs. 69.6% of controls (PR = 1.22). The lack of evaluation of eye movements in standard clinical practice, particularly saccades, may contribute to the under and delayed diagnosis of PSP.

Our study shows that many patients had other diagnostic codes in their medical records that are common misdiagnoses of PSP, occurring before their first PSP diagnostic code. This suggests the likelihood of misdiagnosis given the spectrum of PSP (15) and, in particular, the overlapping symptoms of early Parkinson's disease and early PSP, as well as the lack of knowledge of diagnosing physicians (12). In the present study, Parkinson's disease, depression/bipolar disorder, and dementia were common diagnoses made in PSP patients before their index date. Over half (54.6%) of PSP cases had a diagnosis of Parkinson's disease at some point in the 5 years before first PSP diagnostic code. One other study based on data from an electronic medical record database had similar results reporting that, of the patients who had at least one instance of PSP in their medical record (*n* = 1,335), almost half (46%; 596/1291) had a diagnosis code for Parkinson's disease in the 5 years before their first PSP claim (38). In a study of autopsy-confirmed PSP cases (*n* = 122) from centers in the United Kingdom, Germany, Sweden, Canada, Spain, and the United States, 23% were initially diagnosed with Parkinson's disease (12). The fact that diagnostic codes for PD were frequent among PSP patients in this study may indicate that some of these patients met criteria for the PSP-P subtype.

In the present study, neurologists were most likely to record the first code for PSP, followed by acute care hospitals, and family practice physicians. This suggests that approximately half of PSP patients in the real-world are first diagnosed by a neurologist and only a small proportion are diagnosed by family practice physicians.

Identifying the early signs of PSP can help with early diagnosis, appropriate management, better planning for patients and caregivers, and potential inclusion into research such as registries and clinical trials to potentially slow disease progression and improve understanding of the disease. Early diagnosis is important for this disabling disorder characterized by marked decline in motor function and quality of life, and postdiagnosis survival of ~7 years (2). It may facilitate appropriate disease management, including symptomatic relief, supportive care, and prevention of complications, and use of advance directives and other end of life planning for patients and caregivers, an approach increasingly seen with atypical parkinsonism disorders (39, 40). In this study, in the 5 years before diagnosis many of the hallmark signs of PSP appear to be documented in patients' medical records.

A strength of this study is the use of prospectively collected diagnostic codes through a retrospective nested case-control study design. It is possible that the availability of observational data antedating the first PSP diagnosis may be more reliable than asking patients to recount their medical history, which is common in PSP case-control studies and subject to recall bias. The diagnoses identified in this study can be examined, both separately and in combination, to attempt to develop an algorithm to identify patients at risk of PSP before diagnosis. The clinical features of patients observed before diagnosis, can be considered potential variables in the development of this type of predictive model using large databases, such as the one in this study.

There are several limitations of this study. First, we were unable to confirm the presence of PSP or other disorders clinically because diagnosis was made entirely based on diagnostic codes. Moreover, variability in assessments is an inherent limitation of administrative data given that providers may document claims for billing purposes in different ways. Patients aged >65 years were more likely to be underrepresented in this analysis because the database primarily includes patients with employer-based health insurance plans, potentially excluding some older patients with Medicare (and Medicaid) plans, resulting in a possible underestimation of PSP prevalence. Additionally, there was no distinct billing code for PSP prior to ICD-10 (operational after October 1, 2015); before then, PSP was primarily documented using the ICD-9 code 333.0 for “other degenerative disease of the basal ganglia.” In this study, PSP patients were required to have two or more ICD-10 codes and/or one or more ICD-9 code and one or more ICD-10 code. Therefore, we did not include patients with only one ICD-9 code. This approach potentially reduced the risk of misclassification of PSP cases, but it is possible that some patients included in the study, whether diagnosed by ICD-9 or ICD-10 codes, were not true cases. This is also possible because, although ICD-10 code G23.1 is primarily used to code for PSP, it may also be used for other conditions such as supranuclear, progressive ophthalmoplegia. This is the first known study that examined PSP in a claims database; we know of no validated algorithms for identifying PSP patients in claims data, however, we applied a common methodology for disease identification in claims databases. It is common practice for studies to require two or more diagnostic codes to eliminate “rule-out” cases [see (41) as an example]. We applied the same methodology, and used both ICD-9 and ICD-10 codes to increase sensitivity to detect PSP cases.

In a study by Anderson et al. (38), PSP patients were identified in an electronic-health record (EHR) database by requiring one or more instances of terms related to PSP in the patient's records. The authors identified 1,335 PSP patients who met the study criteria out of ~40 million total patients. This resulted in a larger number of PSP patients, as compared to the present study, however, there is likely a potential risk of misclassification due to the requirement of only one term related to PSP in the patient's record. In contrast, in the present study, which was set in a claims database, we applied a more specific case definition requiring two or more diagnostic codes for PSP in order to increase the likelihood of identifying true PSP patients. Similar to the present study where 54.6% of PSP patients have a PD diagnosis code prior in the 5 years to PSP index date, Anderson et al. found that 46% of PSP patients in their sample had a PD diagnosis in the 5 years prior to first PSP encounter. Further studies of PSP should be conducted in claims and EHR databases to continue to work on the development of optimal algorithms for identifying patients with this rare disease.

In conclusion, this real-world study provides a preliminary estimate of PSP prevalence in the United States and describes the burden of disease in PSP patients before and at the time of diagnosis. Although this study likely underestimates the prevalence of all PSP, it provides valuable new data on the occurrence of PSP in the United States. The data suggest that PSP is probably diagnosed many years after onset, as evidenced by the documentation of symptoms in PSP cases several years before diagnosis. The documentation of possible PSP symptoms in the 5 years before diagnosis, specifically Parkinson's disease, gait abnormalities, pain in joint, fracture, falls and dysarthria provides a better understanding of the difficulties in diagnosing PSP and clinical features/diagnoses that may be informative for development of predictive models of PSP using the diagnostic codes and medications identified in this study. Furthermore, improving our understanding of PSP in real-world data may provide an opportunity for intervention if a disease-modifying therapy becomes available.

## Data Availability Statement

The data analyzed in this study is subject to the following licenses/restrictions: The dataset was generated from an administrative claims database for which Biogen holds an in-house license. Requests to access these datasets should be directed to SE (susan.eaton@biogen.com).

## Author Contributions

MJ, LL, HK, and SE conducted or contributed to the statistical analysis. All authors contributed to conception and design of the study, manuscript revision, and read and approved the submitted version.

## Conflict of Interest

EV, TD, LL, HK, SE, SH, and JK are/were employees of and hold shares/share options in Biogen. IL is supported by the National Institutes of Health grants: 5P50AG005131-33, 2R01AG038791-06A, U01NS090259, 1U54 NS 092089, U01NS100610, U01NS80818, R25NS098999, P20GM109025; Parkinson Study Group, Michael J Fox Foundation, Lewy Body Association, AbbVie, Biogen and Roche. She was member of a Lundbeck Advisory Board and participated in a symposium organized by Sunovion. She receives her salary from the University of California San Diego and as Chief Editor of Frontiers in Neurology. MJ was a contract employee of Biogen and is presently employed by MJ Analytics Ltd. GH received research support from GE Healthcare and Neuropore; has ongoing research collaborations with Orion and Prothena; serves as a consultant for AbbVie, AlzProtect, Asceneuron, Biogen, Biohaven, Lundbeck, Novartis, Roche, Sanofi, UCB; received honoraria for scientific presentations from AbbVie, Biogen, Roche, Teva, UCB, and Zambon; and holds a patent on PERK Activation for the Treatment of Neurodegenerative Diseases (PCT/EP2015/068734).
