# Supplementary material for: Clinical Features of Patients With Progressive Supranuclear Palsy in an US Insurance Claims Database
Source: Front Neurol. 2021 Jun 17;12:571800. doi: 10.3389/fneur.2021.571800 (PMC8245849; doi:10.3389/fneur.2021.571800)
Supplement: Supplementary file 1 [file Data_Sheet_1.docx]

Supplementary Material

## Supplementary Figures

**SUPPLEMENTARY TABLE 1.** Provider type at first PSP diagnostic code

| Provider Type, n (%) | Patients With PSP  n = 630 |
| --- | --- |
| Neurology | 308 (44.3) |
| Acute care hospital | 131 (18.8) |
| Family practice | 77 (11.1) |
| Internal medicine | 34 (4.9) |
| Multispecialty physician group | 26 (3.7) |
| Other facility | 20 (2.9) |
| Psychiatry | 11 (1.6) |
| Emergency medicine | 9 (1.3) |
| Ophthalmology | 9 (1.3) |
| Medical doctor | 8 (1.2) |
| Home health organization/agency | 8 (1.2) |
| Radiology | 6 (0.9) |
| Cardiovascular disease/cardiology | 6 (0.9) |
| Geriatric medicine | 5 (0.7) |

| Supply center | 4 (0.6) |
| --- | --- |
| Extended care facility | 3 (0.4) |
| Pathology | 3 (0.4) |
| Surgeon | 3 (0.4) |
| Rehabilitation facilities | 2 (0.3) |
| Hospice facility | 2 (0.3) |
| Orthopedic surgery | 2 (0.3) |
| Physician assistant | 2 (0.3) |
| Laboratory | 2 (0.3) |
| Other | 14 (2.0) |

Percentages out of all provider types at first visit. A patient may have >1 provider type at first visit.

N=54 provider visits where provider type was missing or unknown.

PSP, progressive supranuclear palsy.

**SUPPLEMENTARY TABLE 2.** Frequency of diagnoses prior to the index date, by time period (overall and years 1–2) in PSP cases (n = 590) and controls (n = 1220)

|  | Time Period Prior to Index Date | | | | | | | | |
| --- | --- | --- | --- | --- | --- | --- | --- | --- | --- |
|  | Any Time^a^ | | | 1 Day to 12 Months^b^ | | | 13 to 24 Months^d^ | | |
| Diagnosis | PSP Cases  n = 590 | Controls  n = 1220 | PR | PSP Cases  n = 477 | Controls  n = 969 | PR | PSP Cases  n = 394 | Controls  n = 780 | PR |
| Movement disorders and related conditions |  |  |  |  |  |  |  |  |  |
| Parkinson's disease | 322 (54.6%) | 12 (1.0%) | 55.5* | 271 (56.8%) | 10 (1.0%) | 55.1* | 124 (31.5%) | 3 (0.4%) | 81.8* |
| Secondary parkinsonism | 72 (12.2%) | 0 (0.0%) | . | 46 (9.6%) | 0 (0.0%) | . | 20 (5.1%) | 0 (0.0%) | . |
| Corticobasal degeneration | 7 (1.2%) | 0 (0.0%) | . | 4 (0.8%) | 0 (0.0%) | . | 1 (0.3%) | 0 (0.0%) | . |
| Multisystem degeneration of the autonomic nervous system | 3 (0.5%) | 0 (0.0%) | . | 3 (0.6%) | 0 (0.0%) | . | 0 (0.0%) | 0 (0.0%) | . |
| Extrapyramidal diseases and abnormal movement disorders | 15 (2.5%) | 0 (0.0%) | . | 10 (2.1%) | 0 (0.0%) | . | 2 (0.5%) | 0 (0.0%) | . |
| Amyotrophic lateral sclerosis | 2 (0.3%) | 0 (0.0%) | . | 2 (0.4%) | 0 (0.0%) | . | 0 (0.0%) | 0 (0.0%) | . |
| Striatonigral degeneration | 4 (0.7%) | 0 (0.0%) | . | 4 (0.8%) | 0 (0.0%) | . | 0 (0.0%) | 0 (0.0%) | . |
| Gait/joint conditions |  |  |  |  |  |  |  |  |  |
| Gait abnormalities | 468 (79.3%) | 266 (21.8%) | 3.6* | 373 (78.2%) | 135 (13.9%) | 5.6* | 224 (56.9%) | 85 (10.9%) | 5.2* |
| Pain in joint | 324 (54.9%) | 439 (36.0%) | 1.5* | 179 (37.5%) | 200 (20.6%) | 1.8* | 143 (36.3%) | 159 (20.4%) | 1.8* |
| Pain in limb | 229 (38.8%) | 274 (22.5%) | 1.7* | 78 (16.4%) | 93 (9.6%) | 1.7* | 107 (27.2%) | 115 (14.7%) | 1.8* |
| Muscle weakness | 200 (33.9%) | 86 (7.0%) | 4.8* | 125 (26.2%) | 43 (4.4%) | 5.9* | 67 (17.0%) | 31 (4.0%) | 4.3* |
| Tremor | 131 (22.2%) | 25 (2.0%) | 10.8* | 74 (15.5%) | 12 (1.2%) | 12.5* | 45 (11.4%) | 8 (1.0%) | 11.1* |
| Cervicalgia | 131 (22.2%) | 118 (9.7%) | 2.3* | 73 (15.3%) | 44 (4.5%) | 3.4* | 43 (10.9%) | 38 (2.9%) | 2.2* |
| Ataxia | 51 (8.6%) | 5 (0.4%) | 21.1* | 34 (7.1%) | 1 (0.1%) | 69.1* | 13 (3.3%) | 0 (0.0%) | . |
| Stiffness of joints | 46 (7.8%) | 25 (2.0%) | 3.8* | 22 (4.6%) | 15 (1.5%) | 3.0* | 11 (2.8%) | 5 (0.6%) | 4.4* |
| Restless legs syndrome | 31 (5.3%) | 22 (1.8%) | 2.9* | 21 (4.4%) | 16 (1.7%) | 2.7* | 13 (3.3%) | 4 (0.5%) | 6.4* |
| Torticollis | 8 (1.4%) | 5 (0.4%) | 3.3* | 3 (0.6%) | 1 (0.1%) | 6.1 | 4 (1.0%) | 3 (0.4%) | 2.6 |
| Falls and injuries |  |  |  |  |  |  |  |  |  |
| Falls | 193 (32.7%) | 75 (6.1%) | 5.3* | 138 (28.9%) | 45 (4.6%) | 6.2* | 53 (13.5%) | 19 (2.4%) | 5.5* |
| History of falls | 160 (27.1%) | 30 (2.5%) | 11.0* | 109 (22.9%) | 14 (1.4%) | 15.8* | 48 (12.2%) | 11 (1.4%) | 8.6* |
| Repeated falls | 52 (8.8%) | 4 (0.3%) | 26.9* | 47 (9.9%) | 4 (0.4%) | 23.9* | 3 (0.8%) | 0 (0.0%) | . |
| Fracture | 224 (38.0%) | 109 (8.9%) | 4.3* | 139 (29.1%) | 56 (5.8%) | 5.0* | 76 (19.3%) | 34 (4.4%) | 4.4* |
| Joint dislocation | 49 (8.3%) | 70 (5.7%) | 1.5 | 19 (4.0%) | 31 (3.2%) | 1.3 | 14 (3.6%) | 19 (2.4%) | 1.5 |
| Cognitive and behavioral conditions |  |  |  |  |  |  |  |  |  |
| Alzheimer's disease | 64 (10.8%) | 25 (2.0%) | 5.3* | 46 (9.6%) | 20 (2.1%) | 4.7* | 17 (4.3%) | 12 (1.5%) | 2.8* |
| Lewy body dementia | 33 (5.6%) | 2 (0.2%) | 34.1* | 23 (4.8%) | 0 (0.0%) | . | 7 (1.8%) | 0 (0.0%) | . |
| Frontotemporal dementia | 30 (5.1%) | 0 (0.0%) | . | 26 (5.5%) | 0 (0.0%) | . | 13 (3.3%) | 0 (0.0%) | . |
| Dementia | 159 (26.9%) | 60 (4.9%) | 5.5* | 124 (26.0%) | 41 (4.2%) | 6.1* | 70 (17.8%) | 22 (2.8%) | 6.3* |
| Memory loss | 182 (30.8%) | 45 (3.7%) | 8.4* | 94 (19.7%) | 20 (2.1%) | 9.6* | 73 (18.5%) | 19 (2.4%) | 7.6* |
| Depression/bipolar disorder | 185 (31.4%) | 139 (11.4%) | 2.8* | 119 (24.9%) | 73 (7.5%) | 3.3* | 84 (21.3%) | 51 (6.5%) | 3.3* |
| Anxiety/phobia | 142 (24.1%) | 142 (11.6%) | 2.1* | 94 (19.7%) | 71 (7.3%) | 2.7* | 56 (14.2%) | 48 (6.2%) | 2.3* |
| Personality disorder | 16 (2.7%) | 2 (0.2%) | 16.5* | 4 (0.8%) | 0 (0.0%) | . | 7 (1.8%) | 0 (0.0%) | . |
| Homicidal and suicidal ideations | 5 (0.8%) | 5 (0.4%) | 2.1 | 2 (0.4%) | 2 (0.2%) | 2.0 | 0 (0.0%) | 1 (0.1%) | . |
| State of emotional shock and stress | 3 (0.5%) | 2 (0.2%) | 3.1 | 1 (0.2%) | 0 (0.0%) | . | 0 (0.0%) | 2 (0.3%) | . |
| Nervousness | 2 (0.3%) | 1 (0.1%) | 4.1 | 2 (0.4%) | 1 (0.1%) | 4.1 | 1 (0.3%) | 0 (0.0%) | . |
| Restlessness and agitation | 2 (0.3%) | 3 (0.2%) | 1.4 | 2 (0.4%) | 3 (0.3%) | 1.4 | 0 (0.0%) | 0 (0.0%) | . |
| Demoralization and apathy | 1 (0.2%) | 0 (0.0%) | . | 0 (0.0%) | 0 (0.0%) | . | 0 (0.0%) | 0 (0.0%) | . |
| Excessive crying | 1 (0.2%) | 0 (0.0%) | . | 0 (0.0%) | 0 (0.0%) | . | 1 (0.3%) | 0 (0.0%) | . |
| Emotional lability | 1 (0.2%) | 1 (0.1%) | 2.1 | 1 (0.2%) | 0 (0.0%) | . | 0 (0.0%) | 1 (0.1%) | . |
| Speech disorders |  |  |  |  |  |  |  |  |  |
| Speech disturbances | 141 (23.9%) | 12 (1.0%) | 24.3* | 94 (19.7%) | 6 (0.6%) | 31.8* | 34 (8.6%) | 2 (0.3%) | 33.7* |
| Voice disturbance | 62 (10.5%) | 18 (1.5%) | 7.1* | 37 (7.8%) | 8 (0.8%) | 9.4* | 22 (5.6%) | 3 (0.4%) | 14.5* |
| Dysarthria | 83 (14.1%) | 4 (0.3%) | 42.9* | 48 (10.1%) | 2 (0.2%) | 48.8* | 18 (4.6%) | 2 (0.3%) | 17.8* |
| Aphasia | 31 (5.3%) | 6 (0.5%) | 10.7* | 21 (4.4%) | 4 (0.4%) | 10.7* | 12 (3.0%) | 1 (0.1%) | 23.8* |
| Apraxia | 21 (3.6%) | 4 (0.3%) | 10.9* | 18 (3.8%) | 3 (0.3%) | 12.2* | 5 (1.3%) | 1 (0.1%) | 9.9* |
| Vision problems |  |  |  |  |  |  |  |  |  |
| Diplopia | 56 (9.5%) | 7 (0.6%) | 16.5* | 36 (7.5%) | 2 (0.2%) | 36.6* | 15 (3.8%) | 3 (0.4%) | 9.9* |
| Subjective visual disturbances | 5 (0.8%) | 2 (0.2%) | 5.2* | 2 (0.4%) | 1 (0.1%) | 4.1 | 0 (0.0%) | 0 (0.0%) | . |
| Other irregular eye movements | 5 (0.8%) | 0 (0.0%) | . | 3 (0.6%) | 0 (0.0%) | . | 2 (0.5%) | 0 (0.0%) | . |
| Eyelid dysfunction | 4 (0.7%) | 0 (0.0%) | . | 2 (0.4%) | 0 (0.0%) | . | 1 (0.3%) | 0 (0.0%) | . |
| Saccadic eye movements | 2 (0.3%) | 0 (0.0%) | . | 2 (0.4%) | 0 (0.0%) | . | 1 (0.3%) | 0 (0.0%) | . |
| Blepharospasm | 2 (0.3%) | 0 (0.0%) | . | 1 (0.2%) | 0 (0.0%) | . | 0 (0.0%) | 0 (0.0%) | . |
| Urinary |  |  |  |  |  |  |  |  |  |
| Urinary disorder | 225 (38.1%) | 243 (19.9%) | 1.9* | 115 (24.1%) | 90 (9.3%) | 2.6* | 95 (24.1%) | 94 (12.1%) | 2.0* |
| Urinary tract infection | 168 (28.5%) | 202 (16.6%) | 1.7* | 101 (21.2%) | 97 (10.0%) | 2.1* | 53 (13.5%) | 66 (8.5%) | 1.6* |
| Other |  |  |  |  |  |  |  |  |  |
| Sleep disorder | 188 (31.9%) | 195 (16.0%) | 2.0* | 116 (24.3%) | 114 (11.8%) | 2.1* | 92 (23.4%) | 82 (10.5%) | 2.2* |
| Fatigue | 294 (49.8%) | 263 (21.6%) | 2.3* | 171 (35.8%) | 105 (10.8%) | 3.3* | 119 (30.2%) | 85 (10.9%) | 2.8* |
| Cerebrovascular disease | 269 (45.6%) | 200 (16.4%) | 2.8* | 176 (36.9%) | 100 (10.3%) | 3.6* | 122 (31.0%) | 76 (9.7%) | 3.2* |
| Dysphagia | 190 (32.2%) | 60 (4.9%) | 6.6* | 127 (26.6%) | 28 (2.9%) | 9.2* | 59 (15.0%) | 20 (2.6%) | 5.8* |
| Normal pressure hydrocephalus | 19 (3.2%) | 2 (0.2%) | 19.6* | 12 (2.5%) | 1 (0.1%) | 24.4* | 5 (1.3%) | 0 (0.0%) | . |
| Pick's disease | 6 (1.0%) | 0 (0.0%) | . | 5 (1.0%) | 0 (0.0%) | . | 0 (0.0%) | 0 (0.0%) | . |
| Prion disease | 1 (0.2%) | 0 (0.0%) | . | 1 (0.2%) | 0 (0.0%) | . | 0 (0.0%) | 0 (0.0%) | . |
| Wilson’s disease | 1 (0.2%) | 1 (0.1%) | 2.1 | 1 (0.2%) | 1 (0.1%) | 2.0 | 0 (0.0%) | 0 (0.0%) | . |
| Niemann-Pick disease | 1 (0.2%) | 1 (0.1%) | 2.1 | 1 (0.2%) | 0 (0.0%) | . | 0 (0.0%) | 0 (0.0%) | . |
| Hypoparathyroidism | 1 (0.2%) | 1 (0.1%) | 2.1 | 1 (0.2%) | 1 (0.1%) | 2.0 | 0 (0.0%) | 0 (0.0%) | . |
| Neuroacanthocytosis | 1 (0.2%) | 0 (0.0%) | . | 0 (0.0%) | 0 (0.0%) | . | 0 (0.0%) | 0 (0.0%) | . |
| Paraneoplastic encephalitis | 1 (0.2%) | 0 (0.0%) | . | 0 (0.0%) | 0 (0.0%) | . | 0 (0.0%) | 0 (0.0%) | . |

Showing cases and controls with >1 diagnostic code.

Presenting diagnostic codes in order of descending frequency in cases.

The following conditions were not diagnosed in any PSP cases and are, therefore, not included in the table: Huntington's disease, neurosyphilis, Whipple’s disease, unhappiness, irritability and anger, violent behavior, anhedonia, impulsiveness, impulse disorder, hostility, and limitation of activities due to disability.

^a^Patients with ≥30 days of continuous follow-up time prior to the index date; **^b^**enrolled in the 1 day to 12 months prior to the index date; **^c^**enrolled in the 13 to 24 months prior to the index date.

*Statistically significant PR.

PR, prevalence ratio; PSP, progressive supranuclear palsy.

**SUPPLEMENTARY TABLE 3.** Frequency of diagnoses prior to the index date, by time period (years 3–5) among PSP cases (n = 590) and controls (n = 1220)

|  | Time Period Prior to Index date | | | | | | | | |
| --- | --- | --- | --- | --- | --- | --- | --- | --- | --- |
|  | 25–36 Months^a^ | | | 37–48 Months^b^ | | | 49–60 Months^d^ | | |
| Diagnosis | PSP Cases  n = 303 | Controls  n = 583 | PR | PSP Cases  n = 188 | Controls  n = 358 | PR | PSP Cases  n = 60 | Controls  n = 121 | PR |
| Movement disorders and related conditions |  |  |  |  |  |  |  |  |  |
| Parkinson's disease | 61 (20.1%) | 1 (0.2%) | 117.4* | 25 (13.3%) | 0 (0.0%) | . | 6 (10.0%) | 0 (0.0%) | . |
| Secondary parkinsonism | 9 (3.0%) | 0 (0.0%) | . | 2 (1.1%) | 0 (0.0%) | . | 1 (1.7%) | 0 (0.0%) | . |
| Corticobasal degeneration | 2 (0.7%) | 0 (0.0%) | . | 0 (0.0%) | 0 (0.0%) | . | 0 (0.0%) | 0 (0.0%) | . |
| Multisystem degeneration of the autonomic nervous system | 0 (0.0%) | 0 (0.0%) | . | 0 (0.0%) | 0 (0.0%) | . | 0 (0.0%) | 0 (0.0%) | . |
| Extrapyramidal diseases and abnormal movement disorders | 0 (0.0%) | 0 (0.0%) | . | 0 (0.0%) | 0 (0.0%) | . | 1 (1.7%) | 0 (0.0%) | . |
| Amyotrophic lateral sclerosis | 0 (0.0%) | 0 (0.0%) | . | 0 (0.0%) | 0 (0.0%) | . | 0 (0.0%) | 0 (0.0%) | . |
| Striatonigral degeneration | 0 (0.0%) | 0 (0.0%) | . | 0 (0.0%) | 0 (0.0%) | . | 0 (0.0%) | 0 (0.0%) | . |
| Gait/joint conditions |  |  |  |  |  |  |  |  |  |
| Gait abnormalities | 128 (42.2%) | 67 (11.5%) | 3.7* | 54 (28.7%) | 32 (8.9%) | 3.2* | 11 (18.3%) | 6 (5.0%) | 3.7* |
| Pain in joint | 100 (33.0%) | 112 (19.2%) | 1.7* | 45 (23.9%) | 69 (19.3%) | 1.2 | 14 (23.3%) | 27 (22.3%) | 1.1 |
| Pain in limb | 66 (21.8%) | 72 (12.3%) | 1.8* | 33 (17.6%) | 37 (10.3%) | 1.7 | 6 (10.0%) | 14 (11.6%) | 0.9 |
| Muscle weakness | 32 (10.6%) | 12 (2.1%) | 5.1* | 12 (6.4%) | 3 (0.8%) | 7.6* | 4 (6.7%) | 4 (3.3%) | 2.0 |
| Tremor | 26 (8.6%) | 4 (0.7%) | 12.5* | 14 (7.4%) | 5 (1.4%) | 5.3* | 2 (3.3%) | 2 (1.7%) | 2.0 |
| Cervicalgia | 28 (9.2%) | 30 (5.1%) | 1.8* | 11 (5.9%) | 17 (4.7%) | 1.2 | 3 (5.0%) | 3 (2.5%) | 2.0 |
| Ataxia | 4 (1.3%) | 1 (0.2%) | 7.7 | 0 (0.0%) | 1 (0.3%) | . | 1 (1.7%) | 0 (0.0%) | . |
| Stiffness of joints | 8 (2.6%) | 5 (0.9%) | 3.1 | 5 (2.7%) | 2 (0.6%) | 4.8 | 1 (1.7%) | 4 (3.3%) | 0.5 |
| Restless legs syndrome | 6 (2.0%) | 3 (0.5%) | 3.9 | 2 (1.1%) | 2 (0.6%) | 1.9 | 1 (1.7%) | 3 (2.5%) | 0.7 |
| Torticollis | 1 (0.3%) | 2 (0.3%) | 1.0 | 0 (0.0%) | 0 (0.0%) | . | 0 (0.0%) | 0 (0.0%) | . |
| Falls and injuries |  |  |  |  |  |  |  |  |  |
| Falls | 26 (8.6%) | 9 (1.5%) | 5.6* | 13 (6.9%) | 6 (1.7%) | 4.1* | 1 (1.7%) | 1 (0.8%) | 2.0 |
| History of falls | 30 (9.9%) | 4 (0.7%) | 14.4* | 11 (5.9%) | 3 (0.8%) | 7.0* | 0 (0.0%) | 1 (0.8%) | . |
| Repeated falls | 0 (0.0%) | 0 (0.0%) | . | 0 (0.0%) | 0 (0.0%) | . | 0 (0.0%) | 0 (0.0%) | . |
| Fracture | 40 (13.2%) | 20 (3.4%) | 3.9* | 21 (11.2%) | 6 (1.7%) | 6.7* | 5 (8.3%) | 3 (2.5%) | 3.4 |
| Joint dislocation | 8 (2.6%) | 11 (1.9%) | 1.4 | 3 (1.6%) | 4 (1.1%) | 1.4 | 3 (5.0%) | 4 (3.3%) | 1.5 |
| Cognitive and behavioral conditions |  |  |  |  |  |  |  |  |  |
| Alzheimer's disease | 17 (5.6%) | 6 (1.0%) | 5.5* | 8 (4.3%) | 1 (0.3%) | 15.2* | 4 (6.7%) | 0 (0.0%) | . |
| Lewy body dementia | 4 (1.3%) | 1 (0.2%) | 7.7 | 0 (0.0%) | 0 (0.0%) | . | 0 (0.0%) | 0 (0.0%) | . |
| Frontotemporal dementia | 9 (3.0%) | 0 (0.0%) | . | 1 (0.5%) | 0 (0.0%) | . | 1 (1.7%) | 0 (0.0%) | . |
| Dementia | 35 (11.6%) | 9 (1.5%) | 7.5* | 13 (6.9%) | 3 (0.8%) | 8.3* | 3 (5.0%) | 1 (0.8%) | 6.1 |
| Memory loss | 45 (14.9%) | 13 (2.2%) | 6.7* | 16 (8.5%) | 3 (0.8%) | 10.2* | 6 (10.0%) | 1 (0.8%) | 12.1* |
| Depression/dipolar disorder | 52 (17.2%) | 34 (5.8%) | 2.9* | 23 (12.2%) | 22 (6.1%) | 2.0* | 5 (8.3%) | 5 (4.1%) | 2.0 |
| Anxiety/phobia | 35 (11.6%) | 27 (4.6%) | 2.5* | 22 (11.7%) | 13 (3.6%) | 3.2* | 7 (11.7%) | 5 (4.1%) | 2.8 |
| Personality disorder | 2 (0.7%) | 0 (0.0%) | . | 3 (1.6%) | 1 (0.3%) | 5.7 | 1 (1.7%) | 1 (0.8%) | 2.0 |
| Homicidal and suicidal ideations | 0 (0.0%) | 0 (0.0%) | . | 2 (1.1%) | 1 (0.3%) | 3.8 | 0 (0.0%) | 0 (0.0%) | . |
| State of emotional shock and stress | 2 (0.7%) | 0 (0.0%) | . | 1 (0.5%) | 0 (0.0%) | . | 0 (0.0%) | 0 (0.0%) | . |
| Nervousness | 0 (0.0%) | 0 (0.0%) | . | 0 (0.0%) | 0 (0.0%) | . | 0 (0.0%) | 0 (0.0%) | . |
| Restlessness and agitation | 0 (0.0%) | 0 (0.0%) | . | 0 (0.0%) | 0 (0.0%) | . | 0 (0.0%) | 0 (0.0%) | . |
| Demoralization and apathy | 0 (0.0%) | 0 (0.0%) | . | 0 (0.0%) | 0 (0.0%) | . | 0 (0.0%) | 0 (0.0%) | . |
| Excessive crying | 0 (0.0%) | 0 (0.0%) | . | 0 (0.0%) | 0 (0.0%) | . | 0 (0.0%) | 0 (0.0%) | . |
| Emotional lability | 0 (0.0%) | 0 (0.0%) | . | 0 (0.0%) | 0 (0.0%) | . | 0 (0.0%) | 0 (0.0%) | . |
| Speech disorders |  |  |  |  |  |  |  |  |  |
| Speech disturbances | 16 (5.3%) | 2 (0.3%) | 15.4* | 3 (1.6%) | 0 (0.0%) | . | 2 (3.3%) | 0 (0.0%) | . |
| Voice disturbance | 7 (2.3%) | 3 (0.5%) | 4.5* | 4 (2.1%) | 1 (0.3%) | 7.6 | 0 (0.0%) | 0 (0.0%) | . |
| Dysarthria | 9 (3.0%) | 0 (0.0%) | . | 2 (1.1%) | 0 (0.0%) | . | 1 (1.7%) | 0 (0.0%) | . |
| Aphasia | 9 (3.0%) | 0 (0.0%) | . | 0 (0.0%) | 0 (0.0%) | . | 2 (3.3%) | 0 (0.0%) | . |
| Apraxia | 2 (0.7%) | 1 (0.2%) | 3.9 | 0 (0.0%) | 1 (0.3%) | . | 0 (0.0%) | 0 (0.0%) | . |
| Vision problems |  |  |  |  |  |  |  |  |  |
| Diplopia | 7 (2.3%) | 1 (0.2%) | 13.5* | 3 (1.6%) | 0 (0.0%) | . | 2 (3.3%) | 0 (0.0%) | . |
| Subjective visual disturbances | 1 (0.3%) | 0 (0.0%) | . | 0 (0.0%) | 0 (0.0%) | . | 1 (1.7%) | 0 (0.0%) | . |
| Other irregular eye movements | 0 (0.0%) | 0 (0.0%) | . | 0 (0.0%) | 0 (0.0%) | . | 0 (0.0%) | 0 (0.0%) | . |
| Eyelid dysfunction | 1 (0.3%) | 0 (0.0%) | . | 0 (0.0%) | 0 (0.0%) | . | 0 (0.0%) | 0 (0.0%) | . |
| Saccadic eye movements | 0 (0.0%) | 0 (0.0%) | . | 0 (0.0%) | 0 (0.0%) | . | 0 (0.0%) | 0 (0.0%) | . |
| Blepharospasm | 0 (0.0%) | 0 (0.0%) | . | 0 (0.0%) | 0 (0.0%) | . | 0 (0.0%) | 0 (0.0%) | . |
| Urinary |  |  |  |  |  |  |  |  |  |
| Urinary disorder | 62 (20.5%) | 71 (12.2%) | 1.7* | 31 (16.5%) | 37 (10.3%) | 1.6 | 4 (6.7%) | 15 (12.4%) | 0.5 |
| Urinary tract infection | 31 (10.2%) | 40 (6.9%) | 1.5 | 30 (16.0%) | 23 (6.4%) | 2.5* | 4 (6.7%) | 6 (5.0%) | 1.3 |
| Other |  |  |  |  |  |  |  |  |  |
| Sleep disorder | 51 (16.8%) | 55 (9.4%) | 1.8* | 22 (11.7%) | 31 (8.7%) | 1.4 | 5 (8.3%) | 11 (9.1%) | 0.9 |
| Fatigue | 74 (24.4%) | 59 (10.1%) | 2.4* | 30 (16.0%) | 42 (11.7%) | 1.4 | 10 (16.7%) | 7 (5.8%) | 2.9 |
| Cerebrovascular disease | 62 (20.5%) | 50 (8.6%) | 2.4* | 30 (16.0%) | 27 (7.5%) | 2.1* | 9 (15.0%) | 4 (3.3%) | 4.5* |
| Dysphagia | 33 (10.9%) | 11 (1.9%) | 5.8* | 18 (9.6%) | 1 (0.3%) | 34.3* | 5 (8.3%) | 0 (0.0%) | . |
| Normal pressure hydrocephalus | 2 (0.7%) | 1 (0.2%) | 3.9 | 4 (2.1%) | 1 (0.3%) | 7.6 | 0 (0.0%) | 0 (0.0%) | . |
| Pick's disease | 1 (0.3%) | 0 (0.0%) | . | 0 (0.0%) | 0 (0.0%) | . | 0 (0.0%) | 0 (0.0%) | . |
| Prion disease | 0 (0.0%) | 0 (0.0%) | . | 0 (0.0%) | 0 (0.0%) | . | 0 (0.0%) | 0 (0.0%) | . |
| Wilson’s disease | 0 (0.0%) | 0 (0.0%) | . | 0 (0.0%) | 0 (0.0%) | . | 0 (0.0%) | 0 (0.0%) | . |
| Niemann-Pick disease | 0 (0.0%) | 1 (0.2%) | . | 0 (0.0%) | 1 (0.3%) | . | 0 (0.0%) | 0 (0.0%) | . |
| Hypoparathyroidism | 0 (0.0%) | 0 (0.0%) | . | 0 (0.0%) | 0 (0.0%) | . | 0 (0.0%) | 0 (0.0%) | . |
| Neuroacanthocytosis | 0 (0.0%) | 0 (0.0%) | . | 1 (0.5%) | 0 (0.0%) | . | 0 (0.0%) | 0 (0.0%) | . |
| Paraneoplastic encephalitis | 0 (0.0%) | 0 (0.0%) | . | 0 (0.0%) | 0 (0.0%) | . | 1 (1.7%) | 0 (0.0%) | . |

Showing cases and controls with ≥1 diagnostic code.

Presenting diagnostic codes in order of descending frequency in cases.

The following conditions were not diagnosed in any PSP cases and are, therefore, not included in the table: Huntington's disease, neurosyphilis, Whipple’s disease, unhappiness, irritability and anger, violent behavior, anhedonia, impulsiveness, impulse disorder, hostility, and limitation of activities due to disability.

**^a^**Patients with PSP enrolled in the 25–36 months prior to index date; ^b^37–48 months prior to index date; ^c^49–60 months prior to index date.

*Statistically significant PR.

PR, prevalence ratio; PSP, progressive supranuclear palsy

**SUPPLEMENTARY FIG. 1.** Patient attrition program. ICD-10, International Statistical Classification of Diseases, 10th revision; ICD-9, 9th revision International Statistical Classification of Disease


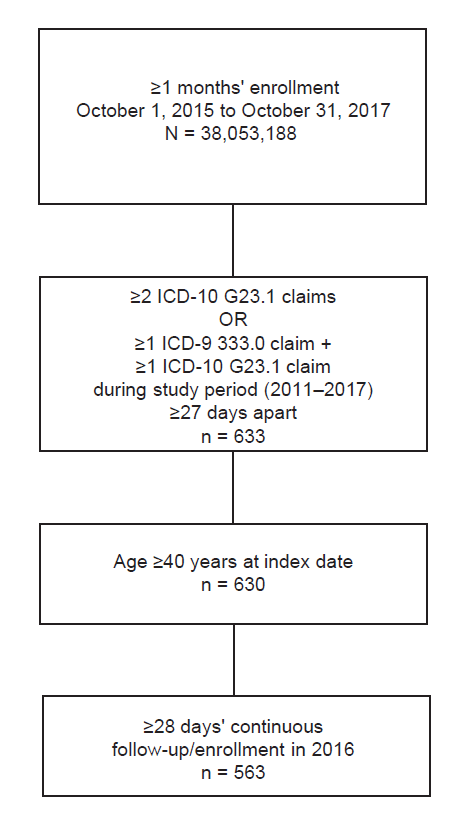


**SUPPLEMENTARY FIG. 2.** Frequency of clinical features suggestive of PSP in cases and controls in the 5 years before the index date. Patients with PSP where ^a^PSP, n = 60; control, n = 121. ^b^PSP, n = 188; control, n = 358. ^c^PSP, n = 303; control, n = 583. ^d^PSP, n = 394; control, n = 780. ^e^PSP, n = 477; control, n = 969. d, day; mo, month; PSP, progressive supranuclear palsy.
